# Childhood Immune Thrombocytopenia: Long-term Follow-up Data Evaluated by the Criteria of the International Working Group on Immune Thrombocytopenic Purpura

**DOI:** 10.4274/Tjh.2012.0049

**Published:** 2014-03-05

**Authors:** Melike Sezgin Evim, Birol Baytan, Adalet Meral Güneş

**Affiliations:** 1 Uludağ University Faculty of Medicine, Department of Pediatrics, Division of Pediatric Hematology, Bursa, Turkey

**Keywords:** Thrombocytopenia, Long-term survival, children

## Abstract

**Objective:** Immune thrombocytopenia (ITP) is a common bleeding disorder in childhood, characterized by isolated thrombocytopenia. The International Working Group (IWG) on ITP recently published a consensus report about the standardization of terminology, definitions, and outcome criteria in ITP to overcome the difficulties in these areas.

**Materials and Methods:** The records of patients were retrospectively collected from January 2000 to December 2009 to evaluate the data of children with ITP by using the new definitions of the IWG.

**Results:** The data of 201 children were included in the study. The median follow-up period was 22 months (range: 12-131 months). The median age and platelet count at presentation were 69 months (range: 7-208 months) and 19x10^9^/L (range: 1x10^9^/L to 93x10^9^/L), respectively. We found 2 risk factors for chronic course of ITP: female sex (OR=2.55, CI=1.31-4.95) and age being more than 10 years (OR=3.0, CI=1.5-5.98). Life-threatening bleeding occurred in 5% (n=9) of the patients. Splenectomy was required in 7 (3%) cases. When we excluded 2 splenectomized cases, complete remission at 1 year was achieved in 70% (n=139/199). The disease was resolved in 9 more children between 12 and 90 months.

**Conclusion:** Female sex and age above 10 years old significantly influenced chronicity. Therefore, long-term follow-up is necessary in these children.

## Abstract (tr)

**Amaç:** İmmün trombositopeni (ITP), izole trombositopeni ile karakterize çocukluk çağında yaygın görülen bir kanama hastalığıdır. Uluslar arası ITP çalışma grubu (IWG), zorlukların üstesinden gelmek için ITP’de terminolojinin, tanımların ve sürecin standardizasyonu hakkında bir uzlaşı raporu yayınlamıştır.

**Gereç ve Yöntemler:** ITP’li hastalarımıza ait kayıtlar Ocak 2000’den Kasım 2009’a kadar, geriye yönelik olarak, IWG’nin yeni kriterleri kullanılarak değerlendirilmek üzere toplandı.

Bulgular: İki yüz bir çocuğun verileri çalışmaya dahil edildi. Ortanca takip süresi 22 ay (12-131 ay) idi. Başvuru anında ortanca yaş ve trombosit sayısı, sırası ile 69 ay (7-208 ay) ve 19x10^9^/L (1-93x10^9^/L) idi. Hastalığın kronikleşmesi açısından iki risk faktörü saptadık: Kız cinsiyet (OR=2,55, CI=1,31-4,95) ve yaşın 10’dan büyük olması (OR=3,0, CI=1,5-5,98). Hayatı tehdit edici kanama, hastaların 5%’inde (n=9) görüldü. Splenektomi yapılması,7 hastada (3%) gerekti. İlk bir yılda splenektomi yapılan 2 hasta göz ardı edildiğinde, tam remisyon (CR) 70% (n=139/199) hastada görüldü. Hastalık, kronik ITP’li olguların 9’unda (%15; 9/60) daha tanıdan itibaren12 ile 90 ay içerisinde düzeldi.

**Sonuç:** Kız cinsiyet ve yaşın 10’dan büyük olması, kronikleşmeyi belirgin olarak etkiledi. Ancak bu çocuklarda uzun sureli takip gereklidir.

## INTRODUCTION

Immune thrombocytopenia (ITP) is one of the most frequent acquired bleeding diseases in children. It is characterized by destruction of the antibody-sensitized platelets by the reticuloendothelial system and the presence of isolated thrombocytopenia in the absence of splenomegaly [1,2]. In the majority of the children, it is a self-limiting disease with complete recovery of the platelets. However, 20% to 30% of children develop the chronic form of the disease [3]. 

Clinical definition, terminology in evaluating patient characteristics, treatment responses, and outcome in both adults and children with ITP show great variety from one study to another. Therefore, a new revision in children and adults has been made for standardization of terminology, definition, and outcome [4].

The aim of this study was to evaluate the data, and long-term outcome in children with ITP according to the recent International Working Group (IWG) report. 

## MATERIALS AND METHODS

The records of patients diagnosed with ITP from January 2000 to December 2009 at the Department of Pediatric Hematology of Uludağ University Hospital were retrospectively collected. The study was approved by the local ethics committee. The data were evaluated according to the recent consensus report of the IWG on ITP [4]. 

Patients with the following criteria were excluded: 1) thrombocytopenia due to systemic disease or medication, 2) children less than 6 months old, 3) patients with incomplete clinical data. The diagnosis was made with the history, physical examination, complete blood count, and examination of the peripheral blood smear [5]. The diagnosis of ITP and the management, treatment, complications, and outcome of the disease was discussed with each family individually during their outpatient appointments, and written informed consent was obtained from all. 

Age, sex, history of preceding infection and vaccination platelet count, bleeding manifestations, seasonal difference, treatment and treatment response at first presentation were recorded. All children had a minimum of 1 year of follow-up.

Treatment was given to children with either platelet count of less than 10x10^9^/L and/or severe bleeding symptoms. Children with minor and/or mucous bleeding symptoms with platelet count of less than 20x10^9^/L to 30x10^9^/L were closely observed and received treatment on demand [5,6,7]. Prednisone at 3-5 mg kg^-1^ day^-1^ for 3-7 days or intravenous immunoglobulin G (IVIG) at 0.8^-1^ g kg^-1^ day^-1^ were the initial therapeutic options. IWG criteria were used for assessing response to treatment; “complete response” (CR) was defined as platelet count greater than 100x10^9^/L. “Response” was defined as platelet count between 30x10^9^/L and 100x10^9^/L or doubling of the baseline count. Any platelet count lower than 30x10^9^/L or less than doubling of the baseline count was described as “no response”. “Refractory” patients included either those with failed splenectomy or those with either severe ITP or increased risk of bleeding requiring frequent therapeutic intervention [4].

The new terms “newly diagnosed” and “persistent” replaced the previous term “acute” for children diagnosed with ITP within the last 3 months and for cases lasting between 3 and 12 months from diagnosis, respectively. Chronic ITP was defined as persisting thrombocytopenia of less than 100x10^9^/L lasting for more than 12 months [4]. The effects of age at diagnosis in progression to chronic ITP were evaluated by classifying the study group into 3 different age groups [8,9]. The groups were as follows: group 1: between ≥6 and ≤12 months, group 2: between >1 year and ≤10 years, and group 3: >10 years. Autoimmune tests such as antinuclear antibody (ANA) and antiphospholipid antibodies (APAs), direct antiglobulin test, hepatitis B (HBV) and hepatitis C (HCV) viruses, and antigenemia were also tested in children with chronic ITP. Helicobacter pylori was also looked for with the urea breath test in the same group. 

Life-threatening bleeding was defined as intracranial hemorrhage (ICH) and/or severe hemorrhage at any site requiring blood transfusion. The indication of splenectomy and recovery following the operation were separately addressed for each case. 

Two patients had splenectomy within the first 12 months of diagnosis. After excluding these cases, the rates of platelet recovery according to patients’ platelet count were evaluated at the 3rd, 6th, and 12th months of diagnosis. 

Statistical calculations were performed using SPSS 16 for Windows. Normal distribution was tested using the Shapiro-Wilk test. Numerical data and categorical variables were analyzed by the Mann-Whitney U or t-tests and the chi-square test, respectively. The odds ratio (OR) and 95% confidence interval (CI) were used to determine the increased relative risk. The results are reported as median, maximum, and minimum values. Statistical significance was accepted as p<0.05.

## RESULTS

A total of 201 children’s records out of 227 were included in the study. The other 26 children’s records were not eligible for the study due to incomplete data. The median follow-up period for all children was 22 months (range: 12-131 months). 

The median age and platelet count at presentation were 69 months (range: 7-208 months) and 19x10^9^/L (range: 1x109/L to 93x10^9^/L), respectively. Females comprised 54% of the study group (n=108) while males were 46% (n=93). The platelet count and age at diagnosis between the sexes were found to be similar (p>0.05). 

There was no previous history of infection and vaccination for 39% of the children (n=78). History of upper respiratory tract infection, viral exanthemas, and acute gastroenteritis was seen in 50% (n=101), 6% (n=12), and 4% (n=8) of the patients, respectively. Two patients (1%) had a history of recent vaccination (rabies and diphtheria-tetanus-pertussis) 

The most frequent symptoms were petechia and ecchymosis (71%). Thirty-six children (18%) were admitted with epistaxis and/or gum bleeding along with petechia and ecchymosis. Twenty-three patients (11%) had no bleeding manifestations. No significant seasonal fluctuation in the incidence of disease was found (p>0.05). 

Therapy was given to 102 (51%) children at first presentation. The rest (n=99; 49%) were observed according to their clinical symptoms. Initial age and sex did not differ between the treatment and nontreatment arms (p>0.05). IVIG was administered to 66 (65%) children, whereas 36 (35%) received corticosteroids as the first therapeutic choice. The features of the different treatment arms at diagnosis are given in Table 1. Treatment response was found similar for both drugs (p>0.05). Drug-related acute complications were seen in 2 (1%) children. Aseptic meningitis due to IVIG was observed in one of these patients, and the other developed severe tonsillitis following corticosteroid treatment. 

**Figure 1:**
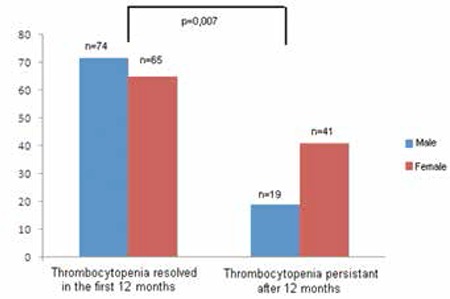
The number of children with persistent versus chronic ITP according to sex.

Within the first 12 months, 2 children required urgent splenectomy. When we excluded these cases, the rest of the patients (30%; n=60) had chronic ITP lasting more than 12 months. Platelet counts and bleeding symptoms at diagnosis, history of preceding infections, and treatment response were found similar between the persistent and chronic groups (p>0.05). However, females had a significantly higher incidence of developing chronic ITP than males (p=0.007) (Figure 1). 

**Figure 2:**
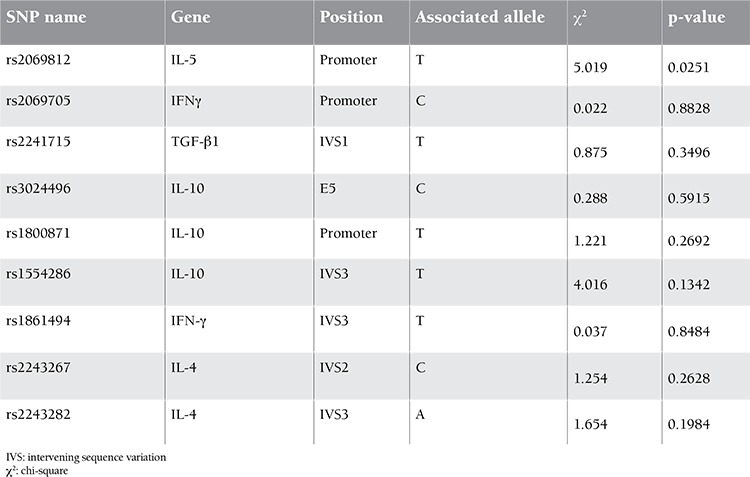
The number of children with persistent versus chronic ITP according to different age groups at diagnosis. *Group 1: between ≥6 and ≤12 months, **Group 2: between >1 year and ≤10 years, ***Group 3: >10 years.

The median age of children with chronic ITP was higher than children with persistent ITP (88 months (range: 10-196 months) and 61 months (range: 7-208 months) respectively, p=0.002). When we evaluated children in 3 different age groups according to their presenting age, children older than 10 years had a significantly higher incidence of developing chronic ITP than the others (Figure 2). 

Among the possible predicting factors for developing chronic ITP, the major predictors were found to be age of more than 10 years old at presentation (OR=3.0, CI=1.5-5.98) and female sex (OR=2.55, CI=1.31-4.95). When we excluded children above 10 years of age, females still had a significantly higher risk of chronicity than males (OR=4.01, CI=1.70-9.50).

**Figure 3:**
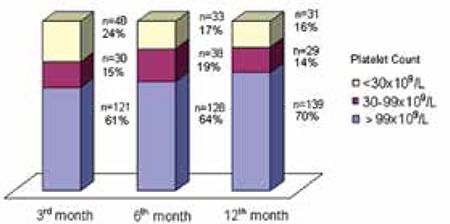
The rate of platelet recovery during 12 months of follow-up (excluding 2 splenectomized patients).

The rate of recovery according to patients’ platelet counts at the 3rd, 6th, and 12th months of diagnosis excluding the 2 splenectomized cases are shown in Figure 3. Eleven (15%) out of 71 children with platelet counts lower than 100x10^9^/L at 6 months achieved CR at 12 months. In total, the platelet count in 139 (70%) out of 199 children gradually rose to normal levels (≥100x10^9^/L) during 12 months of follow-up. 

In addition, platelet recovery subsequently occurred during 20 months (range: 14-90 months) of follow-up in 9 (15%) out of 60 children who were previously defined as “chronic” at 12 months of diagnosis. The sex, age, and initial platelet counts of these children did not differ from those of the nonresponders (p>0.05). 

In total, 15 out of 201 (7.5%) children were refractory, including cases with severe bleeding (n=9) and 6 other children with high risk of bleeding requiring frequent therapy intervention. The characteristics of children with severe bleeding are given in Table 2. Splenectomy had to be performed in 7 of them due to insufficient treatment response to control bleeding symptoms. Splenectomy had to be performed urgently in 2 of them within the first 12 months of diagnosis. CR was achieved immediately in 4 of the 7 splenectomized cases. 

Autoimmune diseases were screened in children with chronic ITP. ANA was found positive in 10% (n=6, 3 females and 3 males) of the children, whereas 8% (n=5) of them had APA positivity. Direct antiglobulin test was also found positive in 3% (n=2) of them without any clinical or laboratory signs of hemolytic anemia. Only one child developed microscopic hematuria. Viral diseases as HBV, and HCV were also screened in 87% (n=52) of the chronic patients, and 4% (n=2) were found positive for the hepatitis B antigen. Eleven out of 60 patients with chronic ITP were screened for H. pylori. Six out of 11 children (67%) were found positive and H. pylori eradication was commenced. None had an increase in platelet count following the eradication.

## DISCUSSION

The IWG in 2009 published a consensus report on standardization of terminology, definition, and outcome criteria in ITP for both adults and children [4]. One of the changes in the criteria of defining ITP was the platelet count; the threshold for diagnosis was established as less than 100x10^9^/L instead of the previously used platelet count of 150x10^9^/L [10,11,12]. 

Another change made by the IWG was in the term “chronic ITP”. The group reserved this term for children with ITP lasting more than 12 months instead of 6 months [4]. Various studies also report that thrombocytopenia resolves in around 70% of children with ITP by 6 months [3,13]. However, complete remission could be achieved in a time longer than this period [14]. The chances of spontaneous remissions are still significant during long-term follow-up, both in adults and children [15,16]. Imbach et al. [2] reported that 25% of children with persistent thrombocytopenia at 6 months had recovered by 12 months. In our study, 11 (15%) out of 71 children with low platelet count at 6 months achieved CR at 12 months (Figure 3). In addition, when the follow-up period was prolonged beyond 12 months, recovery in platelet counts occurred in 9 (15%; n=9/60) more children who were defined as chronic at 12 months. Watts [13] also reported that thrombocytopenia resolved spontaneously in 37 (37%) out of 99 patients with persistent thrombocytopenia between 7 and 96 months from the initial diagnosis. 

The largest study about childhood ITP including 2540 children reported that the mean age and the male/female ratio of the cases of acute and chronic disease were similar [17]. However, it showed that chronic ITP was seen less frequently in infants than in children above 10 years of age [8]. Yaprak et al. [18] from Turkey also reported that children older than 10 years of age had an at least 2-fold increased probability of a chronic outcome. Similar findings were also determined by other reports, revealing that older age is an important predictor of the chronic disease [13,14]. In the current study, we also supported this result, indicating that the risk of developing chronic ITP significantly increased in children older than 10 years of age (OR: 3.0, CI: 1.5-5.98). The other predictor for chronic ITP in our data was female sex. A significant increase of chronicity was noted in females (OR: 2.55, CI: 1.31-4.95). Several studies noted no difference in the incidence of chronic ITP in male versus female patients [13,19]. However, females older than 10 years of age have been reported to develop a more chronic course [6,20,21]. In our study, when we excluded children above 10 years of age, females still had a significantly higher risk of chronicity than males (OR=4.01, CI=1.70-9.50). 

In our cohort, 51% of the patients were treated at diagnosis either with corticosteroids or IVIG. Treatment response did not differ by therapy. Many other studies also supported our finding [13,19]. Various studies from Turkey also reported that the therapy response to prednisolone and IVIG treatments were similar [22,23,24]. The ASH guidelines of 1996 suggested treatment for children with platelet counts of <20x10^9^/L and significant mucous membrane bleeding and for those with counts of <10x10^9^/L and minor purpura [5]. They also recommended hospitalization and treatment intervention in a child with severe, life-threatening bleeding regardless of the platelet counts and for a child with platelet counts of <20x109/L and mucous membrane bleeding. Duru et al. [23] from Turkey also recommended no treatment for children without active bleeding even if they had mucosal bleeding, unless it was continuous and extensive. Current opinion suggests that the treatment decision should be made according to various factors such as the severity of bleeding symptoms, the platelet count, and psychosocial and lifestyle issues [25,26]. The ASH in 2011 also recommended that children without bleeding or with minor bleeding (defined as skin manifestations only, such as bruising and petechia) should be managed with observation alone regardless of their platelet count, but if the child develops an episode of epistaxis that lasts about 15 min, a decision should be made to treat based on the bleeding [27]. Serious bleeding in our group was determined in 5% (n=9) of the children. Various studies also reported that clinically significant bleeding symptoms were observed in between 3% and 6% of children with ITP [6,13,28]. In pediatric literature, ICH incidence ranges from 0.1% to 0.5% [15,29]. ICH occurred only in one child (0.5%). 

In the current study, splenectomy had to be performed in 4% (n=7) of the children (Table 1). Only 2 patients with acute ITP underwent splenectomy within 8 months of diagnosis due to life-threatening bleeding. The rest were chronic cases. Complete remission was achieved in 4 (57%) of 7 splenectomized cases. The recovery rate in the literature varies from 66% to 86% in splenectomized children [30,31]. 

In adults, 1% to 5% of patients with ITP later develop systemic lupus erythematosus (SLE) [33]. In children, 2 main studies evaluated the risk of developing SLE. Hazzan et al. [34] noted that 4% of children (8/222) developed SLE during a mean 4.2 years of follow-up. Zimmerman and Ware [35] suggested a careful follow-up for ANA-positive children with ITP developing autoimmune symptoms. In our study, only one child with ANA positivity developed microscopic hematuria. The role of H. pylori infection in ITP is controversial [26]. The recent guidelines of the ASH do not recommend routine testing for H. pylori in children with persistent and chronic ITP [27]. HIV and HCV screening is recommended in adults upon diagnosis of ITP [26,27]. However, it has been recommended in children if there is a clinical suspicion or high local prevalence and no improvement after 3 to 6 months [26]. We also screened HBV in our cohort since Turkey is considered one of the countries with intermediate endemicity in Europe by the World Health Organization [36]. 

In summary, excluding 2 splenectomized cases, 70% (n=139/199) of patients achieved CR within 1 year. In addition, 9 children achieved CR after 1 year. Girls and children older than 10 years old also carry significantly higher risks of developing the chronic form of the disease. 

## CONFLICT OF INTEREST STATEMENT

The authors of this paper have no conflicts of interest, including specific financial interests, relationships, and/or affiliations relevant to the subject matter or materials included.

## Figures and Tables

**Table 1:**
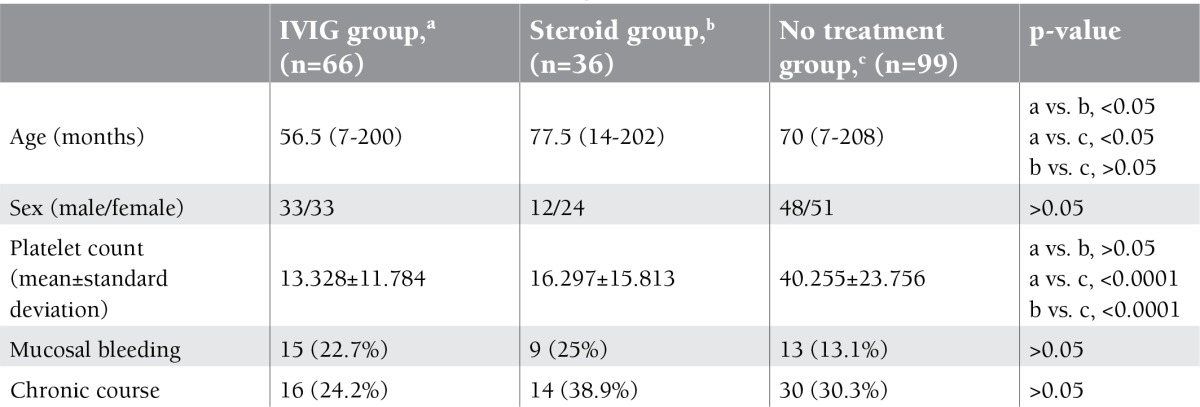
The features of the different treatment arms at diagnosis.

**Table 2:**
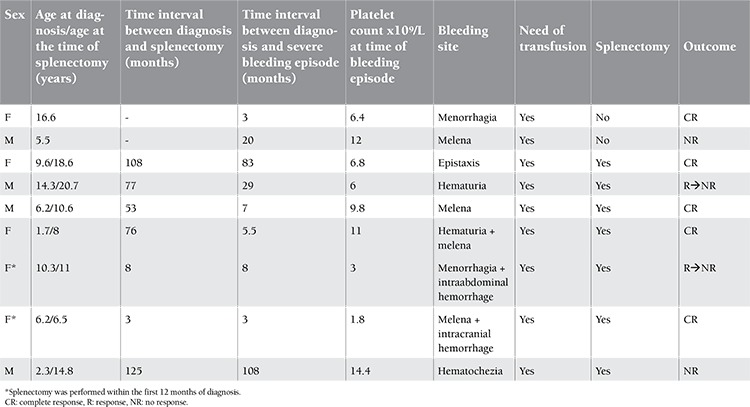
The features of the children with severe bleeding symptoms and characteristics of splenectomized patients.
